# Bi-weekly docetaxel and 5-fluorouracil: an effective and feasible treatment for advanced gastric cancer with poor performance status

**DOI:** 10.1093/gastro/gou051

**Published:** 2014-08-22

**Authors:** Yuting Tan, Wenyun Li, Yonghe Chen, Yanhong Deng, Jian Xiao

**Affiliations:** ^1^Department of Medical Oncology, The Sixth Affiliated Hospital of Sun Yat-sen University, China, ^2^Centre for Quantitative Medicine, Duke-NUS Graduate Medical School, National University of Singapore, Singapore and ^3^Department of Surgery, The Sixth Affiliated Hospital of Sun Yat-sen University, China

**Keywords:** advanced gastric cancer, docetaxel, 5-fluorouracil, poor performance status

## Abstract

**Background:** The survival benefits from cytotoxic chemotherapy have been demonstrated in advanced gastric cancer (AGC). A large proportion of AGC patients initially present poor performance status (PS); however, most of the clinical evidence comes from trials on patients with good PS. A better-designed regimen is greatly needed for AGC patients with poor PS.

**Objective:** To evaluate the efficacy and safety of a modified combination regimen with docetaxel plus 5-fluorouracil (5-FU) every two weeks as first-line treatment in AGC patients with poor PS.

**Methods:** From September 2011 to December 2013, 12 patients diagnosed with AGC with Eastern Cooperative Oncology Group (ECOG) PS scores of 3 or 4 were included in this study. All the patients received docetaxel 60 mg/m^2^ on Day 1, 5-FU 400 mg/m^2^ intravenous (i.v.) bolus on Day 1, and a 46-hour continuous i.v. infusion of 5-FU 2400 mg/m^2^ every two weeks, until disease progressed or patients experienced unacceptable toxicity or declined treatment. Detailed clinical, pathologic and survival data were all recorded.

**Results:** Eleven out of 12 patients were assessable for responses, whereas nine cases (75%) achieved partial response, one (8.3%) achieved stabilized disease, and one (8.3%) had progressive disease. The median progression-free survival was 6.5 months (95% CI: 4.8–8.2). The median overall survival was 12.0 months (95% CI: 9.0–15.0). The most common Grade 3/4 toxicities were anemia in seven patients (58.3%). No patient experienced febrile neutropenia.

**Conclusion:** The novel modification of bi-weekly docetaxel and 5-FU is a promising treatment option for AGC with poor PS, showing great efficacy and acceptable toxicity.

## INTRODUCTION

Gastric cancer is the second leading cause of cancer deaths worldwide [1]. In patients with advanced gastric cancer (AGC) which is unresectable or metastatic, palliative chemotherapy is the only treatment option to improve survival and palliate symptoms [2]. In the past three decades, many clinical trials have been conducted on the survival benefits of cytotoxic agents in different combinations [3, 4]; however, most of these studies only included patients with good performance status (PS). Thus poor PS was always considered as a contraindication for chemotherapy and the best regimen for AGC patients with poor PS is still undetermined.

The Eastern Cooperative Oncology Group (ECOG) PS score is widely used by healthcare professionals, to assess how a patient's disease is progressing, how the disease affects the patient's daily quality of life, and how appropriate treatment and prognosis are determined [5]. Most clinical trials require participants who have an ECOG PS of 0–2; however, AGC patients with ECOG PS 3 or worse are more frequently seen in clinical practice. Investigations on this patients population are of more realistic value.

5-Fluorouracil (5-FU) is a milestone drug for gastrointestinal malignancies. Prolonged infusional 5-FU significantly has increased response rate and substantially reduced the incidence of haematological toxicity when compared with bolus 5-FU [6, 7]. The biochemical modulation of leucovorin also produced superior effect over 5-FU alone [8]. Therefore, the simplified de Gramont regimen [9], which adopts two-day 5-FU infusion with leucovorin, is worthy of investigation on AGC. Docetaxel, a semi-synthetic taxoid, also showed high response rate and prolonged survival in both monotherapy and combination therapy [10, 11]. In addition, the combination of docetaxel and 5-FU analogue could exert potential synergistic actions against human gastric cancer [12]; however, most studies have administered docetaxel every 3 weeks with a high dose intensity that resulted in poor tolerability.

Based on these rationales, we tested a modified bi-weekly regimen of docetaxel and infusional 5-FU in a small patient cohort, to evaluate its efficacy and suitability in AGC patients with poor PS.

## PATIENTS AND METHODS

### Patient selection

This was a pilot study to explore the efficacy and safety of a modified bi-weekly docetaxel and 5-FU regimen as first-line treatment of AGC with poor PS. All the patients were treated in the Sixth Affiliated Hospital of Sun Yat-sen University. Patients who were not eligible for the DaeMon trial (A Phase II Trial Evaluating Biweekly Docetaxel and de Gramont Regimen in First-Line Treatment of Unresectable or Metastatic Gastric Adenocarcinoma. NCT 01567618) were screened for this study. All patients gave informed consent before treatment.

The inclusion criteria were patients with histologically confirmed gastric adenocarcinoma. Patients were 18 years of age or older with ECOG PS scores of 3 or 4, without prior chemotherapy for present lesions. Patients were excluded if there was (i) prior surgery within 3 weeks, radiotherapy within 6 weeks, adjuvant chemotherapy within 12 months or any taxane-containing treatment before entering this trial, (ii) bone-only metastasis, symptomatic brain metastasis, (iii) other simultaneous systemic anticancer treatments or (iiii) concurrent malignant neoplasm.

### Treatment

Patients were to receive docetaxel at a dose of 50 mg/m^2^ over 60 minutes and 5-FU according to simplified de Gramont regimen [400 mg/m^2^ intravenous (i.v.) bolus, followed by 2400 mg/m^2^ 46-hour protracted i.v. infusion] fortnightly. Concomitant dexamethasone from the day before until the day after docetaxel application was administered to prevent fluid retention. Dexamethasone and a 5-hydroxytryptamine Type 3 receptor antagonist were given as anti-emetic prophylaxis before administration of chemotherapy. Prophylactic granulocyte colony-stimulating factor (G-CSF), 300 ug/day, on Days 5–10, was recommended for all treated patients. Treatment was continued in the absence of intolerable side effects, patient refusal to continue or disease progression.

### Evaluation of efficacy and toxicity

Results—categorized as complete response (CR), meaning disappearance of all target lesions; partial response (PR), meaning at least 30% decrease in the sum of the diameters of target lesions); progressive disease (PD), meaning at least 20% increase in the sum of the diameters of target lesions and stable disease (SD), neither sufficient shrinkage to qualify for PR nor sufficient increase to qualify for PD—were evaluated according to Response Evaluation Criteria in Solid Tumours (revised RECIST) 1.1 [13]. Target lesions were assessed every 6 weeks by contrast-enhanced thorax-abdomen-pelvis computed tomography. Objective responses were confirmed by a second evaluation 4–6 weeks later.

Complete blood cell count and serum chemistries were monitored weekly and bi-weekly, respectively. Physical examination and routine laboratory tests were performed before each cycle. Adverse events were graded according to the National Cancer Institute Common Terminology Criteria for Adverse Events version 4 [14].

### Statistical analysis

Continuous variables were summarized as mean ± SD or median (range). Categorical variables were summarized as number (percentage). Progression-free survival (PFS) was defined as the time elapsed from the start of treatment until disease progression or death from any cause, or the date of the last visit. Overall survival (OS) was calculated from the initiation of treatment to death from any cause or the date of the last visit. Data for patients who were progression-free or alive were censored as of the date of the last follow-up visit. Survival curves were estimated with the Kaplan-Meier method. Two-tailed P-value<0.05 was considered to be statistically significant.

## RESULTS

### Patient characteristics

From September 2011 to December 2013, a total of 12 patients were included in this cohort. Three cases (25.1%) were males and nine (75%) were females, with the median age of 47 years old (range: 39–81). Eight patients (66.7%) had PS scores of 3 and four (33.3%) were PS 4 before treatment. Four (33.3%), five (41.7%), and three cases (25%) originated from the antrum, body and oesophago-gastric junction, respectively. All the tumours were poorly differentiated. More than two organs were involved in seven patients (58.3%). Seven (58.3%) patients had received anti-cancer therapy before entering this study (Table 1).

**Table 1.** Patient characteristics and treatment outcomes

Patient No.	Age (years)	Gender	ECOG PS score	Primary tumour site	Histology differentiation	No. of organ involved	Prior treatment	Response	PFS (months)	OS (months)	Disease status
1	80	Male	3	EGJ	poor	2	Untreated	PR	7.1	15.4	Dead of disease
2	63	Female	3	antrum	poor	3	Untreated	PR	7.7	10.7	Alive with disease
3	41	Female	3	antrum	poor	2	Surgery alone	PR	8.0	9.0	Alive with disease
4	44	Female	3	EGJ	poor	2	Adjuvant chemo	SD	6.5	9.2	Dead of disease
5	39	Female	3	body	poor	2	Surgery alone	NA	1.7	3.1	Dead of disease
6	47	Female	3	body	poor	3	Adjuvant chemo	PD	2.2	4.5	Alive with disease
7	43	Female	4	body	poor	3	Untreated	PR	11.7	13.0	Alive with disease
8	55	Male	4	EGJ	poor	3	Surgery alone	PR	10.4	11.4	Alive with disease
9	46	Female	3	body	poor	3	Untreated	PR	6.9	7.2	Alive with disease
10	74	Male	4	antrum	poor	4	Untreated	PR	7.5	8.5	Alive with disease
11	48	Female	3	antrum	poor	2	Untreated	PR	4.5	6.8	Dead of disease
12	42	Female	4	body	poor	3	Untreated	PR	4.0	9.8	Dead of disease

Chemo = chemotherapy, ECOG = Eastern Cooperative Oncology Group, EGJ = oesophago-gastric junction, OS: overall survival, PD = progressive disease, PFS = progression-free survival, PR = partial response, PS = performance status, SD = stable disease.

### Short- and long-term efficacy

**Figure 1.**
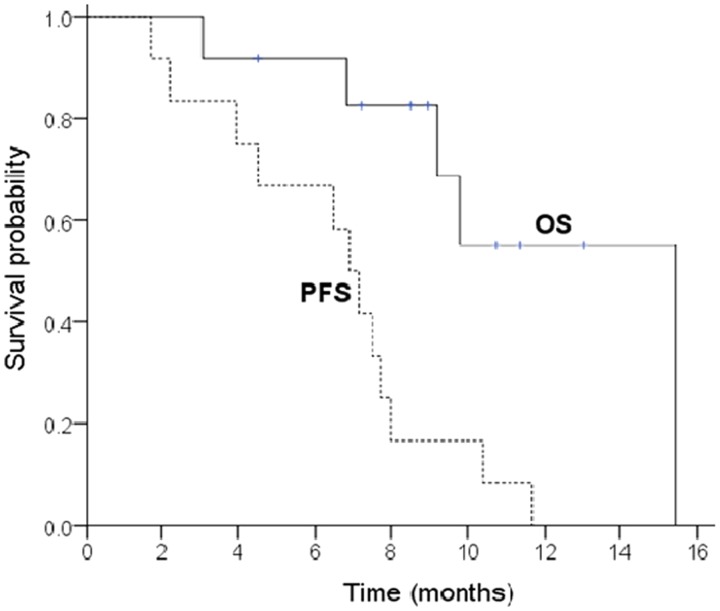
Kaplan-Meier curves of progression-free survival (PFS) and overall survival (OS) for all the patients. The median PFS was 6.5 months (95% CI: 4.8–8.2) and median OS was 12.0 months (95% CI: 9.0–15.0).

Response was assessable in all but one patient, who experienced grade 3 neurotoxicity during the first cycle and stopped treatment. In total, nine patients (75%) achieved PR, one (8.3%) achieved SD, and one (8.3%) had PD. Five out of six patients who presented bowel obstruction caused by peritoneal dissemination, were re-canalized after chemotherapy. With the median follow-up of 9.1 months (3.1–15.4), all the patients had progression on treatment and five (41.7%) died of disease. The median PFS was 6.5 (95% CI: 4.8–8.2) months and the median OS was 12.0 (95% CI: 9.0–15.0) months. The survival curves are shown in Figure 1.

### Toxicity

A total of 70 cycles were administered, with a median of five per patient (range: 1–8). No dose delay or reductions were needed during the treatment. Toxicity was assessable in all the patients. The most common hematological and non-hematological toxicities were anemia (*n = *12; 100%) and alopecia (*n = *11; 91.7%), respectively. Grade 3 or 4 toxicities were anemia (*n = *7; 58.3%), neutropenia (*n = *4; 33.3%), diarrhea (*n = *2; 16.7%), fatigue (*n = *1; 8.3%) and neurotoxicity (*n = *1; 8.3%). More details are listed in Table 2.

**Table 2.** Toxicity

Toxicity (*n = *12)	No. of patients (%)			
	Grade 1	Grade 2	Grade 3	Grade 4
Hematological				
Anemia	1 (8.3)	4 (33.3)	6 (50.0)	1 (8.3)
Leukopenia	8 (66.7)	2 (16.7)	1 (8.3)	0 (0)
Neutropenia	0 (0)	4 (33.3)	4 (33.3)	0 (0)
Febrile neutropenia	0 (0)	0 (0)	0 (0)	0 (0)
Thrombocytopenia	0 (0)	0 (0)	0 (0)	0 (0)
Non-hematological				
stomatitis	2 (16.7)	1 (8.3)	0 (0)	0 (0)
Nausea and vomiting	2 (16.7)	2 (16.7)	0 (0)	0 (0)
Diarrhea	0 (0)	1 (8.3)	2 (16.7)	0 (0)
Constipation	1 (8.3)	0 (0)	0 (0.0)	0 (0)
Fatigue	3 (25.0)	6 (50.0)	1 (8.3)	0 (0)
Alopecia	5 (41.7)	6 (50.0)	NA	NA
Edema	4 (33.3)	0 (0)	0 (0)	0 (0)
Nail change	3 (25.0)	3 (25.0)	0 (0)	NA
Hand foot syndrome	1 (8.3)	1 (8.3)	0 (0)	NA
Liver dysfunction	5 (41.7)	0 (0)	0 (0)	1 (8.3)
Neurotoxicity	0 (0)	0 (0)	1 (8.3)	0 (0)

NA = not applicable.

## DISCUSSION

Chemotherapy has been established as the standard treatment for AGC patients, to prolong survival and improve quality of life; however, patients with poor PS are often excluded from most clinical trials and the treatment options for them were limited. Our study explored a modified bi-weekly regimen of docetaxel and 5-FU in the first-line therapy of AGC patients with poor PS. We found that it was an effective and safe treatment option that achieved a survival benefit similar to that enjoyed by patients with good PS.

Poor PS was traditionally considered as a contraindication for chemotherapy and an index of unfavourable prognosis. For AGC, 1-year OS rate was 43% for patients with PS 0–1, compared with 17% for those with PS 2–3 [15]. Patients with PS of 3 or worse were less likely to prolong survival by chemotherapy and also more likely to experience toxicity. Our results challenged this traditional notion. The median OS of AGC with PS 3–4 was 12 months, which is comparable to, or even better than, previous reports [16]. The reason why patients with poor PS were considered to have a worse prognosis was that healthcare professionals always exclude these patients from consideration for chemotherapy. Our data demonstrated that AGC patients could benefit from regimen of high efficacy and low toxicity.

Our study also showed an excellent toxicity profile. In our study Grade 3 or 4 neutropenia was only seen in four patients (33.3%), which was considered remarkable when compared with the 40.5–66.7% published in previous data [17, 18]. This could be explained by our prophylactic use of G-CSF (300 ug/day for five consecutive days) which was not allowed in the other studies. The relatively low dose intensity of docetaxel in our study, compared with 3-week regimens, might be another important reason for the low incidence of neutropenia. Anemia was the most common Grade 3 or 4 toxicity, seen in seven patients (58.3%) and was significantly higher than the 2–22% reported by previous studies [18-21]. In our study, one-third of patients were PS 4. Poor PS score was usually closely related to low hemoglobin concentration and high incidence of anemia could also be caused by relatively higher dose density of docetaxel. Diarrhea was the most common Grade 3/4 non-hematological toxicity, seen in two patients (7.8%). This could possibly have been caused by the parenteral nutrition needed in patients with poor gastrointestinal function. There was also one patient who experienced Grade 3 neurotoxicity, but similar cases caused by 5-FU have been reported in literature [19].

Our data showed that AGC patients with poor PS achieved a response rate of 75%, which was much higher than those of some trials of the same drug combination with different schedules. In a phase II study, docetaxel (75 mg/m^2^ day 1, every 3 weeks) was used together with continuous-infusional 5-FU (200 mg/m^2^ days 1 through 21, every 3 weeks) leading to a response in 40% of patients [20]. Constenla *et al.* administered docetaxel (75 or 100 mg/m^2^ on Day 1) together with FU (1800 mg/m^2^/24 h on Days 1, 8, and 15) in a 4-week cycle, yielding a response rate of 28% [21]. Our results confirmed that docetaxel in combination with 5-FU is a safe regimen for the treatment of AGC, even in very poor PS patients. This might be partly attributed to the bi-weekly design of our regimen, which shortened the treatment interval and maintained a high dose density. According to the Gompertzian tumour kinetics model, cancer cells grow faster and become more sensitive to chemotherapy between cycles. Thus, a bi-weekly regimen should theoretically be more effective than 3-weekly regimens for the shorter treatment interval.

Our patients had ECGO PS scores of 3–4, indicating shorter expected survival and intolerance of chemotherapy. Our results showed that this modified, dose-sensitive, bi-weekly regimen of docetaxel and 5-FU was efficient and tolerable in AGC in the face of very poor PS; however, we should carefully select patients with impaired PS because they are more vulnerable to the toxicity of chemotherapy. Formal clinical trials are warranted, to focus attention on patients with AGC and poor PS. Our trial could be regarded as an initial exploration in this direction.

**Conflict of interest:** none declared.
